# Prosthetic rehabilitation of segmental mandibulectomy patient using a free fibula flap and Corticobasal implant supported prosthesis: A Case report

**DOI:** 10.1016/j.ijscr.2024.110433

**Published:** 2024-10-10

**Authors:** Syed Akifuddin, Fadia Awadalkreem

**Affiliations:** a Dentomax Centre for Dentistry, Implants and Maxillofacial Surgery, Hyderabad, India; b Department of Prosthodontics, RAK College of Dental Sciences, RAK Medical and Health Sciences University, Ras Al Khaimah, United Arab Emirates

**Keywords:** Case report, Corticobasal implant, Fibula flap, Fixed implant supported prosthesis, Segmental mandibulectomy, Prosthetic rehabilitation

## Abstract

**Introduction:**

Segmental mandibular reconstruction using free fibula flaps and implants is challenging for maxillofacial surgeons and prosthodontists. This case report describes the successful use of Corticobasal implant reconstructive prostheses after a free fibula flap with five years of follow-up.

**Clinical case presentation:**

A 24-year-old female presented to the clinic following segmental defect reconstruction using fibula reconstruction, owing to squamous cell carcinoma resection and seeking prosthetic treatment. An intraoral examination revealed a complete healing of the soft tissue. A panoramic radiograph showed a fibula bone graft rehabilitating the left side of the mandible supporting with a reconstructive plate. A multidisciplinary team was formed. A treatment plan included a mandibular reconstructive fixed Corticobasal implant prosthesis supported by eight basal cortical screw implants (BCS®, Dr. Ihde Dental AG, Switzerland) and a follow-up schedule was formulated.

After 5-years of function, the implant demonstrated a 100 % survival rate, with no implant loss or fracture, excellent peri-implant soft tissue health, complete union of the bone graft, and a very stable prosthesis. The patient reported significant improvements in aesthetics, mastication, phonation, and self-satisfaction.

**Discussion:**

The multidisciplinary team has significantly improved the treatment outcomes. The prescribed treatment modality provides the patient with a fixed treatment modality, immediate loading, reduces the risk of biological and biomechanical complications, and hence improves the patient's functions and satisfaction.

**Conclusion:**

A Corticobasal implant-supported prosthesis can be used in combination with a free fibula flap to reconstruct segmental mandibulectomy patients with a high survival rate and satisfactory aesthetic and functional outcomes.

## Highlights

• Oral rehabilitation following a segmental mandibulectomy presents a challenge.
• A multidisciplinary treatment approach is mandatory to achieve optimum treatment outcomes.
• A free fibula flap can successfully reconstruct the resected mandibular bone and facilitate prosthetic rehabilitation.
• Combining corticobasal implant-supported prostheses with fibula reconstruction has a high survival rate.
• It significantly improves the patient's oral health, aesthetics, mastication, and patient satisfaction.

## 1 Introduction

Mandibular defects have two configurations: marginal and segmental mandibulectomy. In marginal mandibulectomy, the lower border of the mandible is preserved, maintaining both the continuity and the shape of the jaw bone [1]. However, segmental mandibulectomy is associated with a complete resection of the bone, resulting in severe aesthetic disfigurement, impaired functions including mastication, speech, and swallowing, and reducing the patient's self-esteem and quality of life [2].

The amount of hard and soft tissue structure loss, as well as the extension of the defect (anterior arch, lateral segment of the body, or ascending ramus), greatly influences the possibility of patient rehabilitation [3].

Researchers have described several osseocutaneous microvascular free flaps with a variety of donor sites (fibula, iliac crest, radius, metatarsal, rib, and scapula) to reconstruct segmental mandibular defects and maintain jaw continuity with reported superiority of the fibula-free flap [1,4].

The free fibula flap was first described by Hidalgo in 1989 for the reconstruction of mandibular tissue loss [5] with many advantages and a high success rate [[6], [7], [8], [9], [10], [11], [12], [13]], including its long pedicle length, the feasibility of contouring, and the fact that it represents a good site for implant anchorage by providing up to 25 cm of bone stock [5,12,14]. However, some drawbacks have also been reported, including: donor-site morbidities, wound healing problems, compromised graft survival, incomplete reconstruction objectives such as persistent disfigurement, bone resorption, delayed or un-successful bone union [6,12], stress plate fracture [6,12], as well as recurrent infection and traumatic ulcer [6,12] that highlight success as challenging. Many factors should be considered when a patient is selected for a reconstruction procedure, including suitable tissue harvest and microvascular anastomosis, the remaining tissue's quality, quantity, and location, the remaining dentition [9], inter-arch space [9], mouth opening, the tongue and musculature function, the patient's medical status, and a history of radiotherapy and/or chemotherapy [11].

A successful free fibula replaced only the resected bony segment; therefore, the need for prosthetic rehabilitation is still remarkable to retain the patient's state of oral health and function and occlusion using several prosthetic options, including removable and fixed prostheses [[6], [7], [8], [9], [10], [11],^15^]. Today, implant-supported prostheses are considered the gold standard treatment option, with a satisfactory survival rate of 92 % for the implant and 95 % for the graft [6,8].

Corticobasal implants are one-piece implants that have a high success and survival rate in full-ridge support and maxillofacial cases [[16], [17], [18], [19], [20], [21], [22], [23]]. This implant is characterised by a smooth surface, a small penetrating tip, a small diameter, isoelastic properties, and the possibility of splinting, all of which qualify it for use with a fibular reconstructive flap with predictable success [[16], [17], [18], [19], [20], [21], [22], [23]].

This is the first case report that describes the use of Corticobasal implant reconstructive prostheses following a fibula flap with a 5- year function. Ethical approval for the study and informed consents were obtained for treatment and publication. The case was in line with SCARE guidelines [24].

## 2 Presentation of the case

**Fig. 1:**
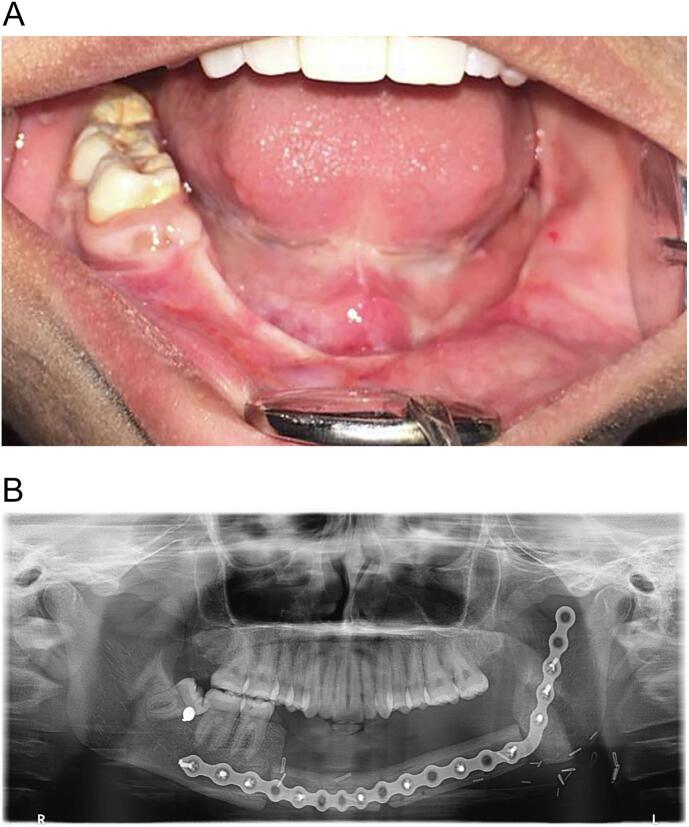
The patient's clinical presentation. A. The intraoral view presents reconstructed mandibular resected bone associated with an obliterated sulcus, complete maxillary teeth, and 46, 47, and 48 teeth. B. The panoramic radiograph reveals a free fibula flap, which has replaced the segmental mandibulectomy, along with a reconstructive plate and a horizontally tilted 48 tooth.

A 24-year-old female presented to the clinic following segmental defect reconstruction using fibula reconstruction, owing to history of Squamous cell carcinoma's resection. An intraoral examination of the patient revealed a complete healing of the soft tissue with considerable interarch space between the jaws (Fig. 1A). A panoramic radiograph (Planmeca Pro Max, Finland) revealed a complete set of maxillary teeth, a bone graft rehabilitating the left side of the mandible's body supported by a reconstructive plate, 46, 47, and a mesial tilted 48 (Fig. 1B). A multidisciplinary team was formed including maxillofacial surgeon with years of experience in Corticobasal implant and Prosthodontist. A treatment plan was formulated, including the use of a mandibular reconstructive fixed Corticobasal implant prosthesis supported with 8 basal cortical screw (BCs, Dr. Ihde Dental AG, Switzerland) implants, surgical extraction of the impacted 48 that had been refused by the patient, and follow-up. The patient had no family or allergy history.

**Fig. 2:**
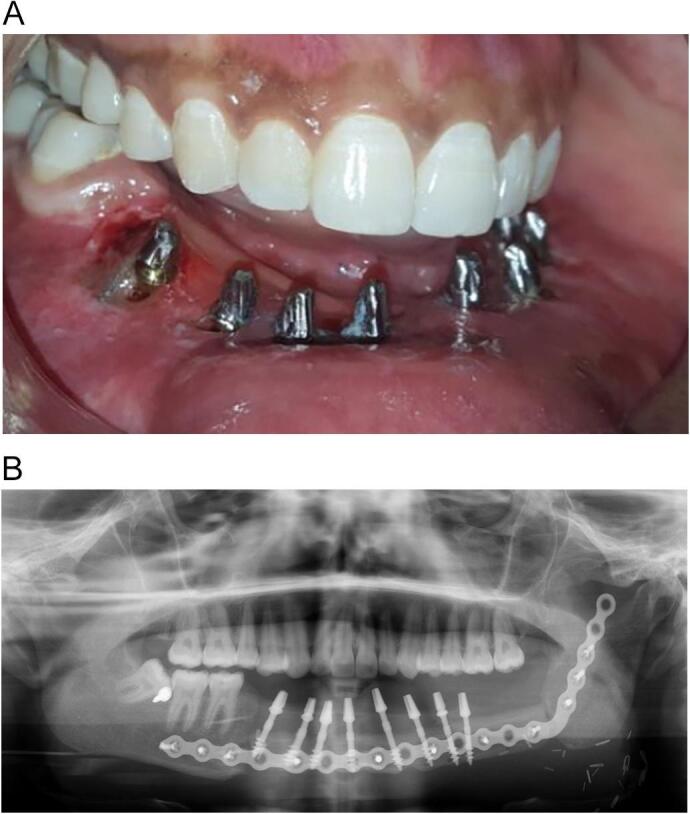
A. An intraoral clinical photograph presenting the distribution of the Corticobasal® implants after impression taken. B. A panoramic radiograph revealing the implant distribution.

A flapless implant osteotomy was performed under an aseptic condition. The patient was asked to rinse with betadine 10 % for 1 min prior to the operation. Local anaesthesia was induced with 2 % lidocaine and epinephrine at 1:100,000. Eight BSC® implants with appropriate lengths and diameters were inserted in the areas 44, 42, 41, 31, 32, 33, 35, and 36 (Fig. 2A). Impression was taken using monophase (VPS; Ivoclar Vivadent AG) following impression copping insertion. Digital panoramic view was taken postoperatively (Fig. 2B). Amoxicillin 1 g antibiotic and Metronidazole 500 mg, and the analgesic, 50 mg diclofenac potassium (Rapidus), were prescribed.

**Fig. 3:**
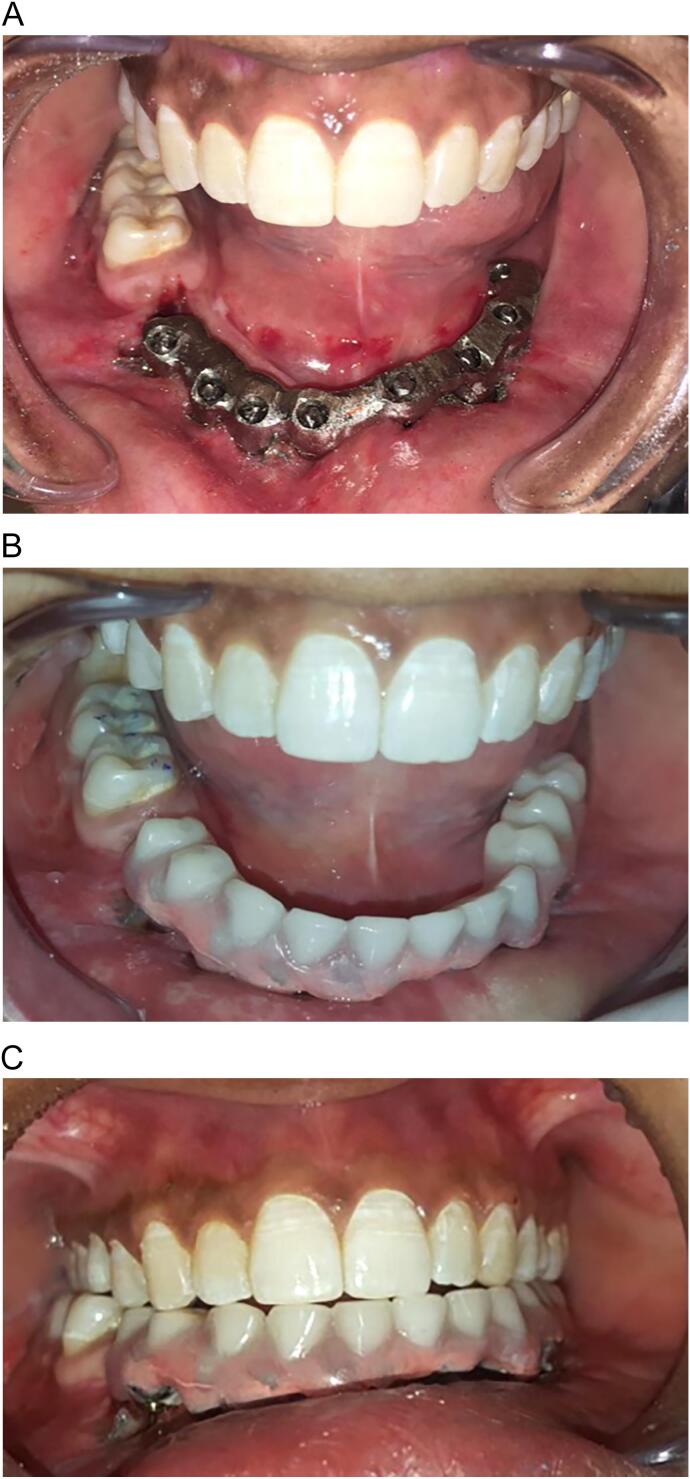
A. The intraoral view showing the metal framework used for implant splinting to distribute the biomechanical load and reduce force per implant. B. The intraoral view reveals the final fixed, immediately loaded, Corticobasal implant-supported prosthesis. C. The intraoral view displays the final fixed, immediately loaded, Corticobasal implant-supported prosthesis, demonstrating its occlusion prior to occlusal adjustment.

One day later, a metal framework was constructed to splint the implants together, and the passive fit of the frame was ensured (Fig. 3A). On the third day, a hybrid acrylic resin prosthesis with an underneath hygienic space was cemented using Fuji cement (GC Corporation, Tokyo, Japan) (Fig. 3B, C). The patient was instructed to use a very soft, small-headed interdental toothbrush to clean the prosthetic and maintain good oral hygiene. Occlusal adjustment had been done, and the patient was scheduled for follow-up after one week, 1,3,6- months, one year, and every year afterward. In each follow-up, the patient should be examined clinically and radiographically.

**Fig. 4:**
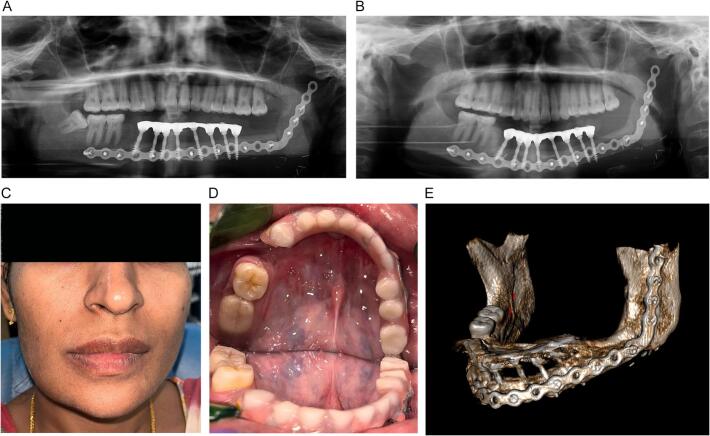
A. The post-operative panoramic radiograph at 6 months of follow-up. (Note: the healing of the flap.) B. The post-operative panoramic radiograph at the one-year follow-up visit (note: the healing of the flap). C. Patient's frontal view at 5-year follow-up. D. The intraoral view of the patient after 5 years of function (the image was taken using a mirror) E. A 3D cone beam CT view showing the complete union of the free fibula flap after 5 years of follow-up.

In the first follow-up, the patient revealed no complaints, exhibited acceptable peri-implant soft tissue health, and had good prosthesis stability. In the 6-month follow-up, an increase in the bone at the area of the bone graft's union was noticed; the patient reported no complaints and significant satisfaction with the treatment outcome concerning aesthetic, phonetic, and mastication. After 5 years of follow-up, the implant showed a 100 % survival rate, with none of the implant being lost or fractured. The patient showed excellent peri-implant soft tissue health, almost complete union of the bone graft, and a very stable prosthesis, with reported improvements in patient oral function and self-satisfaction (Fig. 4A, B, C, D, E).

## 3 Discussion

Rehabilitation of segmental mandibulectomy patients presents a challenge for both the maxillofacial surgeon and prosthodontists [1,8,[18], [19], [20]]. Despite the fact that free vascularizing flaps have a high reported success rate, still failure occurs, highlighting the need for correct intimate collaboration between the different maxillofacial team members and the development of a correct treatment plan [[18], [19], [20]]. In the present case report, a multidisciplinary team ensures the optimum result for the patient in terms of aesthetics, function, and patient satisfaction, as matched by many investigators [8,18,20].

The high primary stability reported in this case matched Ivanjac et al. [22] who documented a higher implant stability quotient values using Resonant Frequency Analysis in comparison to Disk implant in craniomaxillofacial cases. Moreover, the high survival rate in the prescribed case aligns with the reported survival rates of Tahmasebi et al. [6], Navarro Cuéllar et al. [7], who reported implant survival rates of 95 % in fibula flap assisted by virtual surgical planning and 92.6 % with the standard surgical technique, and Land et al. [13], who reported an 88 % survival rate with immediate implantation and 97 % with delayed implantation in cases of free fibula flap following tumor resection [13]. In the same line, Navarro Cuellar et al. [7], Adama [8], Huang et al. [9], and Allen et al. [13] reported the successful use of implant-supported prostheses following free fibula flaps. Additionally, the implant survival rate reported in the case is in accordance with many investigators [[17], [18], [19], [20], [21], [22], [23]]. Lazarov [17] documented a cumulative survival rate of 97.5 % for BCS® after 4 years of function, while Awadalkreem et al. [18] described the successful use of Corticobasal implant-supported prostheses, achieving a 100 % 5-year survival rate in marginal mandibulectomy patients without bone grafting. Gosai et al. [23] reported a 96.8 % survival rate and 3.2 % failure rate with some manageable complications such as: Mobility (2.4 %), 0.8 % pain/discomfort, 0.8 % fracture of abutment at the neck, 9 % prosthesis loosening, and 13 % requirement of relining with no reported incidence of peri-implantitis.

The success of the selected treatment modality can be attributed to the flapless osteotomy technique used for implant insertion as well as the thin implant's penetrating tip, which eliminates the possibility of blood interruption during implant insertion in the grafted area [15]. Moreover, the smooth surface of the implant reduces the susceptibility of plaque adherence to the implant surface and associated peri-implant infection risk, a result that matches the findings of many investigators [[15], [16], [17], [18], [19], [20], [21], [22], [23],^25^]. Furthermore, the monoblock design of the implant as well as the implant splinting using the metal framework ensure better biomechanical distribution of the load, reducing the load transmitted per implant and eliminating the risk of implant overload, as stated by Awadalkreem et al. [20,21], Lazarv et al. [17], Ihde et al. [16], and Misch et al. [25,26]. A result that reduces the risk of plate fracture and mechanical ununion complications in free fibula reconstructive cases and ensures a better treatment outcome that is in line with the prescribed case.

The timing of dental implantation following mandibular construction presents a significant determent of the treatment success. Despite the increasing trend of immediate implantation owing to the fast prosthetic replacement advantage, a higher risk of implant failure may be encountered due to improper implant positioning and susceptibility of tumor recurrence [14]. Hence the delayed implant treatment used in this case is the best treatment option to ensure no recurrence.

Additionally, the significant improvement in the patient's aesthetics, mastication, satisfaction, and improved quality of life matches the observations of Adama et al. [8], Lazarov et al. [16], Awadalkreem et al. [18,20,21], Osman et al. [19], Takaoka et al. [27], Karayazgan-Saracoglu et al. [28], and Yusa et al. [29].

The strength of this study is the high survival rate and significant reported patient satisfaction, while the limitation includes the limited sample size, which highlights the need for a larger study with a larger sample size.

## 4 Conclusion

Within the limitations of the study, the Corticobasal implant-supported prosthesis can be used in combination with a free fibula flap to reconstruct segmental mandibulectomy patients with a high success rate and satisfactory aesthetic and functional outcomes.

## Consent

Written informed consent was obtained from the patient for the research and publication including accompanying images.

## Ethical approval

The research was approved by the he ethical committee of Dentomax Centre for Dentistry and registered at the Research Registry with the unique identifying number: researchregistry10580. https://www.researchregistry.com/browse-the-registry#home/.

## Funding

The authors claim to have no financial interests, either directly or indirectly, in the products or information listed in the article.

## Author contribution

Akifuddin S contributed to the conceptualization, treating the patient, writing, editing, finalization of the case.

Awadalkreem F contributed to the conceptualization, treating the patient, writing, editing, finalization and submission of the case.

## Guarantor

Fadia Awadalkreem.

## Research registration number

The research was registered at the Research Registry with the unique identifying number: researchregistry10580. https://www.researchregistry.com/browse-the-registry#home/.

Written informed consent was obtained from the patients for publication and any accompanying images.

## Conflict of interest statement

The authors declare no conflicts of interest in connection with this research and manuscript.
